# Effects of Hydroxylated Polybrominated Diphenyl Ethers in Developing Zebrafish Are Indicative of Disruption of Oxidative Phosphorylation

**DOI:** 10.3390/ijms18050970

**Published:** 2017-05-03

**Authors:** Jessica Legradi, Marinda van Pomeren, Anna-Karin Dahlberg, Juliette Legler

**Affiliations:** 1 Environment and Health, VU University, 1081 HV Amsterdam, The Netherlands; 2 Institute of Environmental Sciences (CML), Leiden University, 2300 RA Leiden, The Netherlands; m.van.pomeren@cml.leidenuniv.nl; 3 Department of Aquatic Sciences and Assessment, Swedish University of Agricultural Sciences, SE-750 07 Uppsala, Sweden; anna.karin.dahlberg@slu.se; 4 Institute for Environment, Health and Societies, College of Health and Life Sciences, Brunel University, London UB8 3PH, UK; Juliette.Legler@brunel.ac.uk

**Keywords:** zebrafish, OXPHOS disruption, hydroxylated PBDEs

## Abstract

Hydroxylated polybrominated diphenyl ethers (OH-PBDEs) have been detected in humans and wildlife. Using in vitro models, we recently showed that OH-PBDEs disrupt oxidative phosphorylation (OXPHOS), an essential process in energy metabolism. The goal of the current study was to determine the in vivo effects of OH-PBDE reported in marine wildlife. To this end, we exposed zebrafish larvae to 17 OH-PBDEs from fertilisation to 6 days of age, and determined developmental toxicity as well as OXPHOS disruption potential with a newly developed assay of oxygen consumption in living embryos. We show here that all OH-PBDEs tested, both individually and as mixtures, resulted in a concentration-dependant delay in development in zebrafish embryos. The most potent substances were 6-OH-BDE47 and 6′-OH-BDE49 (No-Effect-Concentration: 0.1 and 0.05 µM). The first 24 h of development were the most sensitive, resulting in significant and irreversible developmental delay. All substances increased oxygen consumption, an effect indicative of OXPHOS disruption. Our results suggest that the induced developmental delay may be caused by disruption of OXPHOS. Though further studies are needed, our findings suggest that the environmental concentrations of some OH-PBDEs found in Baltic Sea wildlife in the Baltic Sea may be of toxicological concern.

## 1. Introduction

In recent years, halogenated phenolic compounds (HPCs), such as hydroxylated polybrominated diphenyl ethers (OH-PBDEs), have been found in many marine species [1]. OH-PBDEs are not industrially produced but can be formed via metabolic transformation of anthropogenic polybrominated diphenyl ethers (PBDEs) [2], which have been extensively used as flame retardants and now are widely found in the environment [3]. PBDEs are lipophilic substances which have shown to bioaccumulate and biomagnify in marine food webs [4]. OH-PBDEs can also be produced naturally in the marine environment, for example, by algae and cyanobacteria [5]. Natural production of brominated organic substances provides means for algae to scavenge hydroxide peroxide (H_2_O_2_) [6]. Hydrogen peroxide is produced during photosynthesis and photorespiration but various stress factors such as desiccation, altered salinity, temperature and light can also result in increased production of H_2_O_2_ [7]. Recently, natural production of 2,4,6-tribromophenol (a precursor for naturally synthesised OH-PBDEs) was found to be induced by stress factors such as herbivory, light and salinity in the filamentous macroalgae *C. tenuicorne* [8]. Hence, natural production of OH-PBDEs may be induced by factors altered by changing climate and eutrophication.

Given the relatively hydrophobic characteristics of OH-PBDEs, uptake from water and food could lead to elevated concentrations in fish. The metabolic capacity in fish is not fully understood and might be species specific as some have reported metabolic transformation of PBDEs to OH-PBDEs in pike [9] whereas little or no transformation was observed by others using zebrafish [10,11]. Demethylation of naturally produced methoxylated polybrominated diphenyl ether (MeO-PBDEs) may be an additional pathway for formation of OH-PBDEs in fish. Both zebrafish and Japanese medaka have been shown to metabolically convert 6-MeO-BDE47 to 6-OH-BDE47 [11,12]. Furthermore, for zebrafish and medaka, OH-PBDEs and MeO-PBDEs can be transferred maternally from female fish to their eggs [11,12]. 

OH-PBDEs are detected frequently in the environment and studies have indicated that the toxicity of OH-PBDEs exceeds that of PBDEs [13,14]. Both PBDEs and OH-PBDEs are known for their endocrine disrupting potency, such as estrogenic activity [13]. The presence of a hydroxyl group, however, greatly increases the endocrine disrupting potency [13]. OH-PBDEs and PBDEs are structurally similar to thyroid hormones and thereby have the ability to bind to thyroid hormone receptors and thyroxin transporting molecules (transthyretin), with OH-PBDEs showing a greater affinity than PBDEs [14]. The effects on the thyroid hormone system may also underlie the neurotoxicity of OH-PBDEs and PBDEs, in addition to their more direct toxic effects on the (developing) nervous system and brain [15].

Some of the most sensitive effects of OH-PBDEs have been shown in studies from our laboratory, which have demonstrated that these substances are very potent disruptors of oxidative phosphorylation (OXPHOS) in zebrafish embryonic fibroblast (PAC2) cells and extracted zebrafish and rat liver mitochondria [10,16]. Importantly, our research has shown that mixtures of OH-PBDEs at environmentally relevant concentrations result in strong synergistic effects using in vitro models [16]. OXPHOS can be disrupted either via abolishing the link between substrate oxidation and ATP synthesis (i.e., protonophoric uncoupling) or via inhibition of complexes of the electron transport chain. OXPHOS disruption is a well-studied and highly conserved mechanism. Despite the obvious differences between mammal and fish physiologies, properties of mitochondrial respiration are very similar [17]. OXPHOS disruption leads to alterations in mitochondrial membrane potential and oxygen consumption. Treatment of chinook salmon eggs with uncouplers of mitochondrial respiration (disruptors of mitochondrial membrane potential) results in increased oxygen consumption [18]. Whereas, treatment with an inhibitor of oxidative phosphorylation, on the other hand, reduced oxygen (O_2_) consumption to zero [18]. These studies demonstrate that the measurement of oxygen consumption can be indicative of OXPHOS disruption.

Disruption of OXPHOS in fish is also associated with developmental delay [19,20]. Zebrafish embryos reduce their developmental rate and even arrest their development when exposed to OXPHOS disruptors before the stage of midblastula transition [19]. Lai et al. [20] screened 12,000 natural products on effects on early zebrafish development. Metabolic and biochemical assays confirmed that all of the molecules that induced developmental arrest without necrosis also inhibited the electron transport chain. The potency to induce developmental arrest in zebrafish has also been shown for the OH-PBDE congeners, 3-OH-BDE47, 6-OH-BDE47 and 5-OH-BDE47 [21]. Studies in our laboratory have shown that 6-OH-BDE47 exposure induces a delay and subsequent arrest in development at nanomolar concentrations, whereas BDE-47 and 6-MeO-BDE47 showed no toxic effects in embryos or adult zebrafish [10].

Here, we investigate whether OH-PBDEs disrupt oxidative phosphorylation in vivo in fish by exposing zebrafish embryos to a wide range of OH-PBDEs that have been reported in the environment, and monitoring their developmental toxicity. Potential effects on OXPHOS were measured by monitoring oxygen consumption during the first 24 h of development, as well as precise scoring of developmental stage to determine potential effects on the rate of development. An environmental mixture composed of seven OH-PBDEs representative of the concentrations found in Baltic blue mussels (*Mytilus edulis*) was also assessed [22]. The mixture experiment was performed to identify possible mixture effects of compounds that occur together naturally, as well as to confirm previous in vitro results [16]. A recovery experiment was performed to identify whether the induced developmental delay was irreversible. To get a better picture of the actual exposure levels, the exposure concentrations of 6-OH-BDE47 in medium were measured.

## 2. Results

### 2.1. Developmental Toxicity Profile

The developmental toxicity of 17 hydroxylated PBDEs was assessed using zebrafish embryos exposed from 2 to 144 hpf. Most substances induced a delay in development visible as reduced pigmentation and shorter tails compared to the control. At the highest tested concentrations (>1–5 µM), lethality was observed for all substances ). Low effect concentrations (LOEC) were generally in the low µM or nM ranges (Table 1). Although the LOECs after 72 hpf were the same as after 6 days of exposure, the severity of the effects increased with prolonged exposure time. Embryos which showed effects compared to the control (mostly delayed development) at 24 hpf were generally dead at 144 hpf. Embryos that survived until 144 hpf showed malformations such as tail malformations (curved and bend tails) as well as cardiac oedemas, including slower heart beats (Table S1). The least potent substances were 2′-OH-BDE28 and 2′-OH-BDE66 with a No Observed Effect Concentration (NOEC) of 2 and 4 µM, and 6-OH-BDE47 was the most potent with a NOEC of 0.1 µM (Table 1).

### 2.2. Developmental Arrest and Delay 

**Figure 1:**
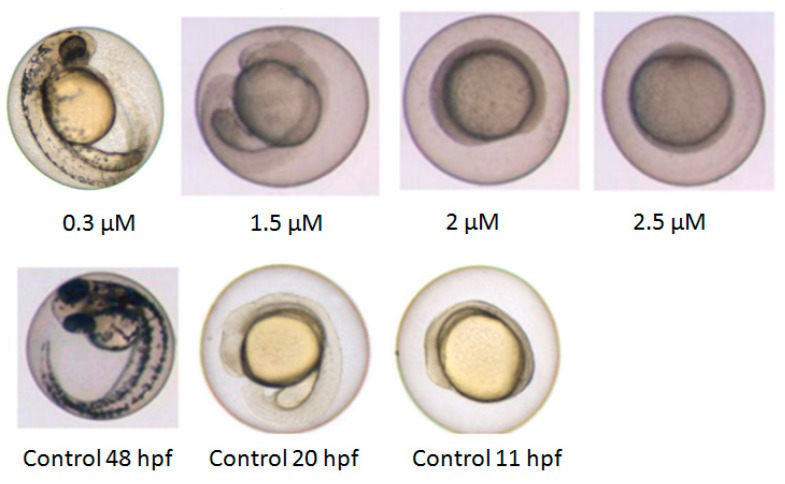
Developmental delay in zebrafish embryos exposed to 2′-OH-6′-Cl-BDE68. Images of 48 hpf old embryos exposed to different concentrations (**upper** row). The delayed embryos look like control embryos at 11 and 20 hpf (**lower** row). Magnification was 2×.

The most encountered effect detected within the toxicity screen was a delay in development. All exposed embryos displayed this within the first 2 days of exposure. In Figure 1, the delay within the first 48 h of development is shown for 2′-OH-6′-Cl-BDE68. The higher the exposure concentration, the slower the development. At 2.5 µM, the embryo was still in the shield stage whereas the control embryo was close to hatching. Interestingly, the embryos exposed to all substances at different concentrations show no other malformations such as oedemas until 48 hpf.

To further investigate the developmental delay, equipotent effect concentrations for a delay in development were identified. The LOEC concentrations shown in Table 1 (24 hpf) all reduce the growth of the embryos. At 24 hpf, exposed embryos looked like 17 hpf old control embryos (7 h delay in development). These concentrations were, on average, four times higher than the LOECs derived from the chronic exposures (144 hpf) shown in Table 1. Again, 2′-OH-BDE28 and 2′-OH-BDE66 were the least potent substances and 6-OH-BDE47 the most potent. If the development during the first 24 h was severely delayed, generally no further development was observed in the following 24 h when the exposure was continued.

**Figure 2:**
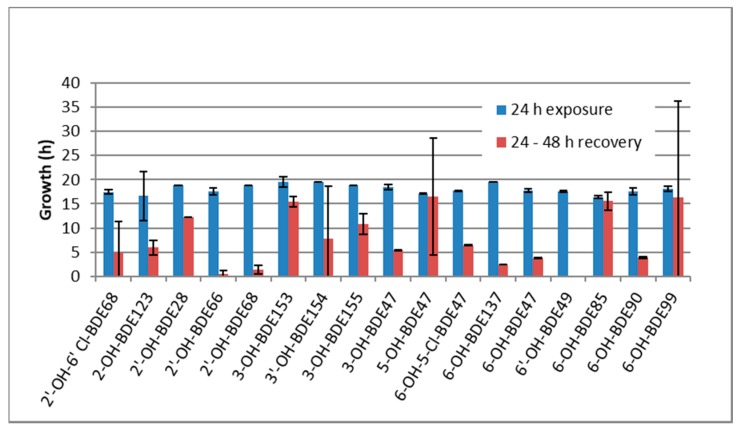
Irreversible developmental delay in zebrafish embryos exposed to hydroxylated polybrominated diphenyl ethers. Development after exposure of zebrafish from 0 to 24 h (blue bars). The red bar presents the development from 24 to 48 hpf after the medium was replaced with clean medium. Concentrations are the LOECs (1 dpf) from Table 1. The error bars are the standard deviation over three replicates.

Furthermore, we performed a recovery experiment. The development could not be recovered by removing the exposure medium and replacing it with embryo standard water. This indicates that the effects on developmental delay are not reversible. Figure 2 shows that most substances even lead to a complete arrest of zebrafish development, as most embryos show nearly no progress in development between 24 and 48 h of exposure (blue and red bars). Some substances, such as 6-OH-BDE85 did not completely arrest development but severely slowed it down.

### 2.3. Oxygen Consumption

**Figure 3:**
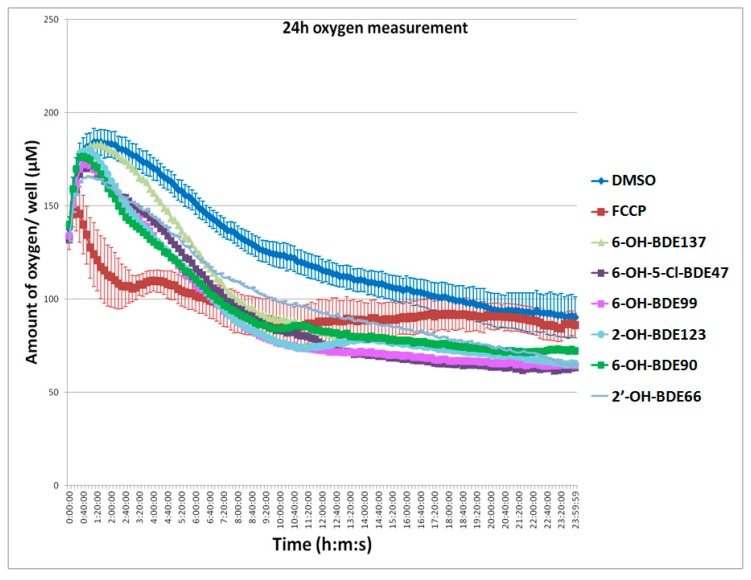
Representative graph showing that hydroxylated polybrominated diphenyl ethers (OH-PBDEs) lead to increased oxygen consumption. Amount of oxygen per well (µM) during the first 24 h of development. Solvent control (DMSO), positive control (Carbonyl cyanide 4-(trifluoromethoxy) phenylhydrazone (FCCP) 0.5 µM) and six different OH-PBDEs at different exposure concentrations (Lowest Observed Effect Concentration (LOECs) for oxygen consumption in Table 1). The error bars present the standard deviation over 12 wells (*n* = 12).

As an indication of OXPHOS disruption, all substances were tested for their effects on oxygen consumption by exposing embryos during the first 24 h of development. As shown in Figure 3, oxygen levels first increased due to the adaptation to the higher temperature in the spectrofluorometer. OH-PBDEs increased the oxygen consumption and lowered the amount of oxygen in the exposure medium compared to the control (Figure 3, Table 1). Control and OH-PBDE exposures displayed first an increase of oxygen in the well plate then a decrease. The increase is caused by the adaption of the temperature in the well plate from room temperature (21 °C) to 26 °C in the spectrofluorometer. The prototypical OXPHOS uncoupler Carbonyl cyanide 4-(trifluoromethoxy) phenylhydrazone (FCCP) (0.5 µM) was included as a positive control and showed the strongest effect leading to a direct reduction of the oxygen content. This reduction was so strong that the temperature adaption increase was not visible anymore. At lower FCCP concentration, this effect was seen. The concentrations by which substance affected oxygen consumption were in the low µM range (Table 1). The 2′-OH-BDE28 and 2′-OH-BDE66 were the least potent substances (LOEC = 6 and 7.5 µM) and 6-OH-BDE47 the most potent (LOEC = 1 µM).

### 2.4. Mixtures

**Figure 4:**
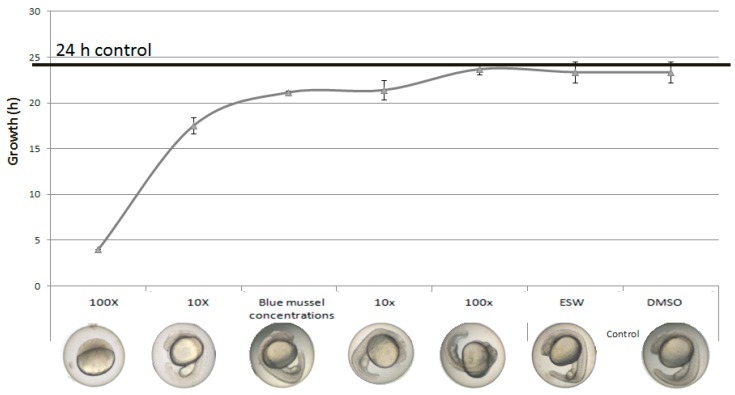
Effects on growth of zebrafish embryos exposed to a mixture of OH-PBDEs for 24 h. The concentrations of the test compounds in the mixture were modelled to represent levels found in Baltic blue mussels, and were tested as concentrated mixtures (10X and 100X) or diluted mixtures (10× and 100×). The error bars show the standard deviation over three replicates.

Zebrafish embryos were exposed to a mixture of seven OH-PBDEs at concentrations found in Baltic blue mussels [22]. In mixtures diluted 10× or more, a visible delay in development within the first 24 h was found (Figure 4). The concentrations used for both the 10× and 1× blue mussel mix were all below the measured NOEC for developmental delay based on individual substances (Table S2). This suggests a clear effect of the mixture that is not observed when the chemicals are tested individually at these concentrations. Severe developmental delay was observed in embryos exposed to a mixture containing 100 times more concentrated levels than the reported levels in blue mussels (Figure 4).

### 2.5. Actual Exposure Concentration

To get a better idea of the exposure concentrations, the exposure medium before exposure, after the first 24 h and after 48 h (24–48 h) was collected and the actual levels of three different 6-OH-BDE47 concentrations measured (Table S3). The concentrations chosen did not induce visible malformations in the embryos. The measured concentration before exposure at t = 0 was around nine times lower than the nominal concentration. After exposure, the concentrations were significantly lower (4.5×–6× lower), suggesting that the compound is taken up into the embryo, or bound to the plastic material of the well plate. The measured concentration from 24 to 48 h was lower than from 0 to 24 h. These results indicate that effect concentrations of 6-OH-BDE47 are even lower than reported here for nominal concentrations.

## 3. Discussion

### 3.1. Hydroxylated Polybrominated Diphenyl Ethers (OH-PBDEs) Developmental Toxicity

In this study, we tested 17 different OH-PBDEs and showed that all induced developmental toxicity. Exposure from 500 nM to 5 µM concentrations from 2 to 144 hpf induced severe developmental effects and lethality. No increase of toxicity from 72 to 144 hpf could be observed. This indicates that the early stages of zebrafish development, prior to 72 hpf, are more sensitive to OH-PBDE exposure than older stages. Interestingly, different effects were observed at different stages of development; after 72 hpf, we observed effects such as decreased movement, lower heartbeats, cardiac oedema, curved tails and less pigmentation. At 24 hpf, all substances induced the same effect, namely a prominent delay in development. No other effects were observed at this stage. The difference in observed effects between the early and later developmental stages might be due to life-stage-specific toxic modes of action that are activated later in development. On the other hand, cardiac oedema and reduced heartbeat are quite commonly observed effects and might also simply reflect a secondary stress response to the effects induced earlier in development. Since our focus was on the developmental delay and oxygen consumption, we might have missed other effects at the molecular level (e.g., thyroid hormone disruption). When comparing LOECs derived from 24 and 144 hpf, a good correlation was found when all effects are included (*R*^2^ = 0.7) and an even stronger correlation (*R*^2^ = 0.9) when tail malformations are excluded (Figure S2). This indicates that effects on tail development might be specific for individual substances as only five out of the 17 substances showed this effect. The other effects observed (developmental delay, cardiac oedema, reduced movement and heart beat) appear to be specific for all OH-PBDEs tested in this study as all substances displayed these effects. The developmental delay seen in the first 24 hpf was concentration dependant. Similarly, Usenko and colleagues showed that 6-OH-BDE47, 3-OH-BDE47, and 5-OH-BD47 delayed development in a concentration dependent manner also at low µM concentrations in zebrafish [21]. The developmental delay observed in our study was generally so severe that a complete developmental arrest was detected. Nearly no additional growth was seen up to 48 hpf, even after removing the exposure solution and replacing it with standard embryo water. It is not clear whether the induced damage in the embryo causes this or the substance remaining in the embryo.

### 3.2. Hydroxylated Polybrominated Diphenyl Ethers (OH-PBDEs) Disrupt Oxidative Phosphorylation in Zebrafish Embryos

Exposure of zebrafish embryos to OXPHOS disruptors before the midblastula transition (~3–5 hpf) is known to reduce their developmental rate and even arrest their development [19,20]. The 6-OH-BDE47 alters the expression of genes involved in proton transport and carbohydrate metabolism and alters the oxygen consumption of extracted mitochondria in zebrafish which might be a sign of OXPHOS disruption [10]. OXPHOS disruption leads to alterations in mitochondrial membrane potential and oxygen consumption in all organisms. In embryos exposed from 2 to 24 hpf, all tested hydroxylated PBDEs were able to change oxygen consumption at low µM concentrations. The concentrations by which OH-PBDEs altered oxygen consumption were, in all cases, slightly higher than those which caused developmental delay. This difference in concentration might be due to the different experimental set up from 24-well plates (developmental delay) and 96-well plates (oxygen consumption). In 96-well plates, the surface–volume ratio is much smaller which might affect oxygen levels in the medium and lead to a reduced sensitivity. Nevertheless, the concentrations by which OH-PBDEs altered oxygen consumption and delayed or arrested development within the first 24 hpf were highly correlated (*R*^2^ = 0.94, Figure S3). Based on *R*^2^ = 0.94, the oxygen consumption is a more sensitive endpoint than the visual developmental delay. Taken together, the observed developmental delay and related effects on oxygen consumption, both hallmarks of OXPHOS disruption, suggest that hydroxylated PBDEs likely disrupt OXPHOS in developing zebrafish. Furthermore, in another study, we showed that the same hydroxylated PBDEs disrupted OXPHOS in cells and isolated liver mitochondria [16]. In the extracted mitochondria, effect concentrations were between 0.02 and 34 µM and in the cell assay between 5.6 and 100 µM. Interestingly, effects on development of zebrafish embryos at 24 hpf were seen at concentrations about 10× lower than effects in the in vitro assay, indicating how sensitive living zebrafish are to OH-PBDEs. In living zebrafish as well as in in vitro models [16], 6-OH-BDE47 was the most potent OH-PBDE, causing development delay at 0.5 µM, concentrations similar to the model OXPHOS disruptor FCCP. 

The tested OH-PBDEs cover substances of both natural and anthropogenic origin and a range of pKas and log Kows [16]. No clear correlation between pKa or log Kow and potency of OXPHOS disruption in zebrafish was found. This could also be seen in our previous in vitro study, in which the position (para, ortho, meta) of the hydroxyl group of PBDEs did not correlate with the potencies of OH-PBDEs to disrupt OXPHOS [16]. This suggests that there might be other physical—chemical characteristics of OH-PBDEs or multiple modes of actions (MoAs) which contribute to the potency observed.

### 3.3. Mode of Action (MoA) of OH-PBDEs

This study provides evidence that suggests that OH-PBDEs disrupt OXPHOS. However, there may be additional MoAs involved in the observed effects. Hydroxylated PBDEs are endocrine disruptors and may affect thyroid hormone metabolism [23]. At relatively low levels (10 and 100 nM), 6-OH-BDE47 has been shown to significantly upregulate Dio1 mRNA expression at 22 hpf which might affect triiodothyronine (*T*_3_) levels and thereby delay development [24]. Studies have also shown that 6-OH-BDE47 significantly lowers TRβ expression during development [25,26], and affects expression of other nuclear receptors such as AhR [26,27]. Further research is needed to investigate whether hydroxylated PBDEs disrupt OXPHOS directly via interference with the electron transport chain or mitochondrial membrane or whether their interference with the thyroid system or other nuclear receptors plays a role in the observed phenotype.

### 3.4. Environmental Mixture Effects of OH-BDEs

This study showed that a short exposure of 24 h of zebrafish eggs to a mixture of OH-PBDEs at concentrations found in blue mussels from the Baltic Sea resulted in a developmental delay. Importantly, these concentrations show no observable effects when tested as individual substances, suggesting synergistic effects similar to the in vitro study published previously [16]. Effects on development of the mixture were first observed at nominal exposure concentrations 10× lower than measured in blue mussels. Minimal internal concentrations measured in Baltic herring (*Clupea harengus*) from two sites in the Baltic Sea by Dahlberg et al. [28] were between 3 and 1000 times lower than our reported (nominal) LOECs for individual substances, suggesting a narrow margin of exposure for specific substances (Table 2). In perch, even higher levels than in herring were recently reported [8]. These findings suggest that the environmental concentrations of certain OH-PBDEs found in blue mussels, perch and herring in the Baltic Sea may be of toxicological concern. Hence, further studies are needed to investigate how adult and juvenile wildlife are affected by disturbed OXPHOS in the Baltic Sea.

## 4. Material and Methods

### 4.1. Chemicals

2′-Hydroxy-2,4,4′-tribromodiphenyl ether (2′-OH-BDE28); 2′-hydroxy-2,3′,4,4′-tetrabromodiphenyl ether (2′-OH-BDE66); 2′-hydroxy-2,3′,4,5′-tetrabromodiphenyl ether (2′-OH-BDE68); 2-hydroxy-2′,3,4,4′,5-pentabromodiphenyl ether (2-OH-BDE123); 3-hydroxy-2,2′,4,4′-tetrabromodiphenyl ether (3-OH-BDE47); 3-hydroxy-2,2′,4,4′,5,5′-hexabromodiphenyl ether (3-OH-BDE153); 3′-hydroxy-2,2′,4,4′,5,6′-hexabromodiphenyl ether (3′-OH-BDE154); 3-hydroxy-2,2′,4,4′,6,6′-hexabromodiphenyl ether (3-OH-BDE155); 5-hydroxy-2,2′,4,4′-tetrabromodiphenyl ether (5-OH-BDE47); 6-hydroxy-2,2′,4,4′-tetrabromodiphenyl ether (6-OH-BDE47); 6′-hydroxy-2,2′4,5′-tetrabromodiphenyl ether (6′-OH-BDE49); 6-hydroxy-2,2′,3,4,4′-pentabromodiphenyl ether (6-OH-BDE85); 6-hydroxy-2,2′,3,4′,5-pentabromodiphenyl ether (6-OH-BDE90); 6-hydroxy-2,2′,4,4′,5-pentabromodiphenyl ether (6-OH-BDE99); 6-hydroxy-2,2′,3,4,4′,5-hexabromodiphenyl ether (6-OH-BDE137); 2′-hydroxy-6′-chloro-2,3′,4,5′-tetrabromodiphenyl ether (2′-OH-6′-Cl-BDE68); 6-hydroxy-5-chloro-2,2′,4,4′-tetrabromodiphenyl ether (6-OH-5-Cl-BDE47) were synthesized at Stockholm University as described elsewhere [29,30,31]. Carbonyl cyanide 4-(trifluoromethoxy) phenylhydrazone (FCCP) (≥98% purity) was purchased from Sigma-Aldrich (St. Louis, MO, USA). All substances were dissolved in dimethyl sulfoxide (DMSO, Arcos Organics, Geel, Belgium) and stored in darkness at room temperature.

### 4.2. Zebrafish Toxicity Experiments

For the experiments, eggs from wild type zebrafish kept under standard conditions were used [32]. Zebrafish were purchased from Ruinemans, The Netherlands. Fish were put together in the morning in a breading catch after one night separation (males from females). The quality of the clutch was checked using a stereo microscope (M7.5; Leica, Amsterdam, The Netherlands). Before 2 h post fertilization (hpf), eggs (five per well for initial screening and 10 per well for the final experiments) were exposed to the diluted substances in 2 mL of Embryo Standard Water (ESW: 100 mg/L NaHCO_3_, 20 mg/L, KHCO_3_ 180 mg/L MgSO_4_ and 200 mg/L CaCl_2_) at 26 °C in a 24-well plate. For each concentration, three replicates were included. In all experiments, solvent control (DMSO, 0.01%), and negative control (ESW) were incorporated.

All mixture experiments were carried out within the same experiment. All tested concentrations and mixtures had a maximum total DMSO concentration below 0.01%. For the mixture experiment, zebrafish embryos were exposed to a mixture of seven OH-PBDEs at concentrations found in Baltic blue mussels [22] in the same setup as the other exposure experiments (see above). The mixtures were tested in a dilution series, ranging from 100× more concentrated than environmental levels, to 100× more diluted (10×–100×). The used OH-PBDEs and the corresponding concentrations in the mixture are shown in the supplementary Table S2.

Concentration–response experiments with a wide range of concentrations of the substances were performed to assure a monotonic response and to identify the concentrations used in the following experiments For the developmental stage (phenotypical age) assessment, we first staged control embryos by taking pictures at every hour of development from 0 to 48 hpf. We then assessed developing zebrafish in concentration–response experiments for their developmental stage. When the solvent and negative control embryos within an experiment reached the developmental stage of 24 h (Figure S1A) based on a defined control picture, the developmental stage of the exposed embryos was assessed. Images were obtained with a digital camera coupled to a stereomicroscope (M7.5, Leica). For the recovery experiments, the exposure medium was removed from the wells and the embryos washed several times with ESW. The development was assessed again when the control embryos showed a development of 48 hpf (Figure S1B) according to a predefined picture. The developmental stage per replicate was assigned as the stage that >90% of the embryos reached. In most cases, all exposed embryos in the well displayed the same stage.

Chronic exposure was assessed from <4 hpf up to 144 hpf, during which time the test water was refreshed daily with new exposure media (ESW), containing the initial concentration of the substances. Development compared to the control were scored at 24, 48, 72 and 144 hpf. Dead embryos were immediately removed from the test in order to prevent infection by bacteria.

### 4.3. Oxygen Consumption Experiments

A method was established using commercially available 96-well plates that have an oxygen sensitive spot on the bottom of each well allowing for quantitative measurement of the oxygen concentration in the well using a standard spectrofluorometer. Details are described in the manufacturer’s manual. To measure changes in the mitochondrial respiration, eggs were exposed to 300 µL exposure media (ESW and substance) in a 96-well OxoPlate (PreSense, Regensburg, Germany), one egg per well, with *n* = 12 eggs per compound or control. All OH-PBDEs were analysed. Solvent (DMSO, 0.01%) and positive (FCCP, 0.5 μM) controls were incorporated in each 96-well plate. Oxygen level was measured every 10 min for 24 h using a fluorescence reader at 26 °C (SpectraMax Gimini EM Fluorescence reader, Molecular Devices, Sunnyvale, CA, USA) at an excitation wavelength of 540 nm and two emission wavelengths of 590 and 650 nm. Oxygen consumption was calculated as described in the OxoPlate manual [33]. SoftMax Pro 5.2 Software (Molecular Devices, Sunnyvale, CA, USA) was used as the computer program to control the spectrofluorometer.

### 4.4. Measurement of 6-OH-BDE47 Exposure Concentration

#### 4.4.1. Chemicals

Dichloromethane (DCM) and *normal*-hexane (*n*-hx) were purchased from Merck (Darmstadt, Germany) and were both of suprasolv grade. Water (HPLC grade) was purchased from VWR International (Fontenay-sous-Bois, France). Sodium hydroxide (puriss p.a.), Tetrabutylammoniumbisulfate (puriss, ≥99.0%) and Methyl iodide (purum, 99.0%) were purchased from Sigma-Aldrich (Steinheim, Germany). An aqueous solution of tetrabutylammonium (TBA), a phase transfer catalyst, was prepared from tetrabutylammonium bisulfate (0.5 M, 100 mL). The solution was extracted with *n*-hx (100 mL) before adding Sodium hydroxide (4.1 g) to form tetrabutyl ammonium hydroxide (TBAOH, 0.5 M, 100 mL). TBA was chosen as it has been previously used as a phase transfer for extractive acetylation of phenols with pentafluorobenzoyl chloride (PFBCl) [34].

The 4′-Hydroxy-2,3′,4,5′,6-pentabromodiphenyl ether (4′-OH-BDE121) was used as surrogate standard and synthesized in house [35]. The 2,2′,3,4,4′,5′-Hexabromodiphenyl ether (BDE-138) was used as volumetric standard and purchased from Wellington Laboratories, Inc. (Guelph, ON, Canada). The 6-Methoxy-2,2′,4,4′-tetrabromodiphenyl ether (6-MeO-BDE47) was synthesized in house [31] and used as authentic reference standard.

#### 4.4.2. Extraction and Derivatization

The exposure media were transferred to 4 mL glass vials and stored frozen (−20 °C) until analysis. Prior to extraction, each sample was thawed and weighed. The 4′-OH-BDE121 (5 ng) was added as surrogate standard to each sample before being transferred to test tubes. DCM (4 mL) was added to each empty sample vial and ultrasonicated for 30 min as a washing step. The sample volume in each test tube was adjusted to 2 mL with water (HPLC-grade) before adding the phase transfer catalyst TBAOH (0.5 M, 2 mL). Each sample was vortexed (30 s) before adding the DCM (4 mL) from the sample vial. The empty (and dried) sample vials were then weighed. The samples were partitioned (10 min) and centrifuged (3 min, 3000 rpm). For each sample, the organic phase was transferred to a new test tube and the water phase re-extracted with DCM (4 mL), as described. The organic phases were pooled in the new test tubes and evaporated to 3 mL under a gentle stream of nitrogen gas. Methyl iodide (0.3 mL) was added to each sample and ultrasonicated for 1 h and placed in darkness at room temperature for 1 h. The samples were then gently evaporated to near dryness using nitrogen gas and the solvent changed to *n*-hx (4 mL). To clean the sample from iodine ions which could obstruct the instrumental analysis, each sample was partitioned with 4 mL of water (HPLC-grade) for 10 min. After centrifugation (3 min, 3000 rpm) the organic phase was transferred to a new test tube. The remaining water phase was re-extracted with *n*-hx (4 mL), as described. The organic phases were pooled and evaporated to 0.5 mL using nitrogen gas. Each sample was spiked with BDE-138 (5 ng) as volumetric standard and transferred to the vial prior to instrumental analysis.

#### 4.4.3. Instrumental Analysis

All samples were analyzed on a gas chromatograph (Agilent Technologies 7890A, Santa Clara, CA, USA) equipped with an electron capture detector (ECD) and an autosampler (Agilent Technologies 7693). The injector was operated in splitless mode at 250 °C and the analytes were separated on a non-polar BPX5 GC capillary column (15 m × 0.25 mm i.d., 0.1 µm; SGE Analytical Science, Ringwood, VIC, Australia) using helium as carrier gas. The temperature program for the column oven was 80 °C (held for 1 min), 30 °C/min to 170 °C, 10 °C /min to 230 °C followed by 5 °C/min to 300 °C (held for 1 min). The detector temperature was 325 °C and nitrogen gas was used as makeup gas.

#### 4.4.4. Quality Assurance/Quality Control

For each batch of samples, a procedural solvent blank was prepared in parallel as quality control to ensure the absence of background contamination. The limit of detection (LOD) was set to three times the signal noise ratio (S/N = 3) and the limit of quantification as three times the LOD. The mean recovery of the added surrogate standard (4′-OH-BDE121) was 88% (RSD 21%), which shows that the analytical method worked satisfactorily for analysis of OH-PBDEs.

### 4.5. Data Analysis and Statistics

The linear regression and the Pearson product–moment correlation coefficient were both calculated using Excel software (2007). Significant effects on oxygen consumption were determined using an unpaired Student’s *t*-test comparing the amount of oxygen in the well after 10 h exposure, where a difference (*p* < 0.05) from the corresponding 96-well plate control was considered significant. At 10h, an increase in oxygen consumption as well as a decrease compared to the DMSO control can be identified. The kinetics of the curves were not analysed. The analysis of the recorded measurements was performed using Excel software (2007). For malformations, the NOEC levels showed <5% effect to the control and the LOEC ≥5% effect compared to the control. For the developmental delay, the LOEC was defined as a >7 h delay of development compared to the control. Bellow 7 h, it was not possible to clearly identify a delay due to too minor differences.

## 5. Conclusions

Our results indicate that OH-PBDEs found in the environment are toxic to developing zebrafish at low µM levels. The induced effects seemed to be related to the disruption of OXPHOS. The link between the endocrine disrupting potential and the capability to disrupt OXPHOS needs to be further investigated. The synergistic mixture effects seen in vivo and the increasing number of studies reporting OH-PBDEs in different environmental samples including humans highlight the importance of such research. 

## Figures and Tables

**Table 1:** Effect concentrations of hydroxylated polybrominated diphenyl ethers (OH-PBDEs) for developmental delay after 24 h; malformations after 72 and 144 hpf; and increased oxygen consumption after 24 h. All nominal concentrations are in µM.

	Developmental Delay		Malformations and Mortality		
	24 hpf		72 hpf/144 hpf		Significant Increase in Oxygen Consumption
Compound	NOEC *	LOEC ^&^	NOEC **	LOEC ^&&^	LOEC ^†^
2′-OH-6′-Cl-BDE68	0.3	1.5	0.75	1	1.5
2-OH-BDE123	1.25	2	0.5	1	2.5
2′-OH-BDE28	5.25	6	2	3	6.5
2′-OH-BDE66	6	7	4	5	7.5
2′-OH-BDE68	0.5	1	0.4	0.6	1.75
3-OH-BDE153	3	3.5	0.5	1	4.75
3′-OH-BDE154	2	2.5	0.5	1	3.25
3-OH-BDE155	4	5	0.5	1	7
3-OH-BDE47	3.5	5	1	2	6.5
5-OH-BDE47	2	2.5	1	2	2.75
6-OH-5-Cl-BDE47	0.9	1.2	0.5	0.75	1.2
6-OH-BDE137	0.1	1.2	0.2	0.5	2
6-OH-BDE47	0.1	0.5	0.1	0.5	1
6′-OH-BDE49	0.05	0.75	0.1	0.5	2.5
6-OH-BDE85	0.5	1	0.5	0.75	1.25
6-OH-BDE90	0.25	2	0.5	1	2.5
6-OH-BDE99	0.5	1	0.5	1	2

* NOEC: No Effect Concentration, defined as the highest concentration at which the stage of the embryo was the same as the control; ^&^ LOEC: Low Effect Concentration, defined as the concentration at which the embryo was staged at 17 hpf, whereas the control embryos were staged at 24 hpf; ** NOEC: concentration at which <5% effect was found (similar to controls); ^&&^ LOEC: lowest concentration at which >5% effect was found; ^†^ LOEC: lowest concentration at which oxygen consumption was statistically different from the solvent control (*p* < 0.05). OH-BDE = hydroxylated brominated diphenyl ether.

**Table 2:** Comparison of effect concentrations and measured concentrations (µM) in Baltic herring (*Clupea harengus*) from two sites in the Baltic Sea by Dahlberg et al. [28]. LOD = limit of detection.

All in µM	144 hpf		Environmental Measured Fish Concentration			Margin of Exposure
Compound	NOEC	LOEC	Location: Askö	Location: Ängskärsklubb	Minimum	LOEC/Minimum
2-OH-BDE123	0.5	1	0.002	<LOD	0.002	500
2′-OH-BDE68	0.4	0.6	0.01	0.007	0.01	60
6-OH-BDE137	0.2	0.5	<LOD	<LOD	<LOD	not applicable
6-OH-BDE47	0.1	0.5	0.2	0.2	0.2	3
6-OH-BDE90	0.5	1	0.001	0.002	0.001	1000
6-OH-BDE99	0.5	1	0.01	0.2	0.01	100

## Supplementary Materials

Supplementary materials can be found at www.mdpi.com/1422-0067/18/5/970/s1.
