# Supplementary material for: Effects of Hydroxylated Polybrominated Diphenyl Ethers in Developing Zebrafish Are Indicative of Disruption of Oxidative Phosphorylation
Source: Int J Mol Sci. 2017 May 3;18(5):970. doi: 10.3390/ijms18050970 (PMC5454883; doi:10.3390/ijms18050970)
Supplement: Supplementary file 1 [file ijms-18-00970-s001.pdf]

Supplementary Materials: Effects of Hydroxylated Polybrominated Diphenyl Ethers in Developing Zebrafish Are Indicative of Disruption of Oxidative Phosphorylation

Jessica Legradi, Marinda van Pomeren, Anna-Karin Dahlberg and Juliette Legler

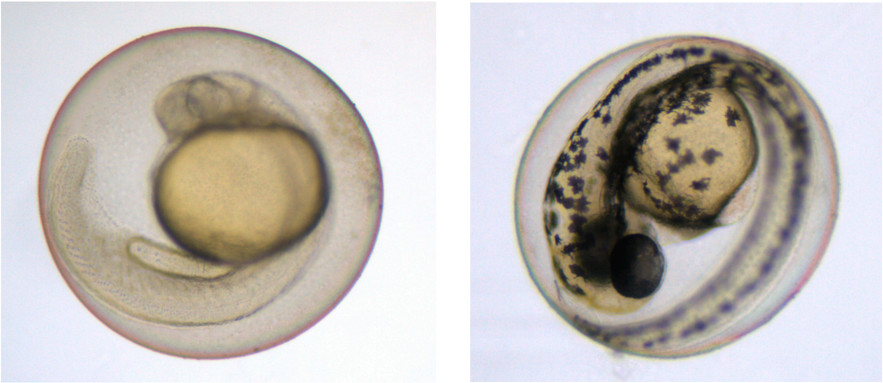

(**A**) (**B**)

Figure S1. (**A**) Control image of 24 hpf; (**B**) Control image of 48 hpf. Magnification was 2×.

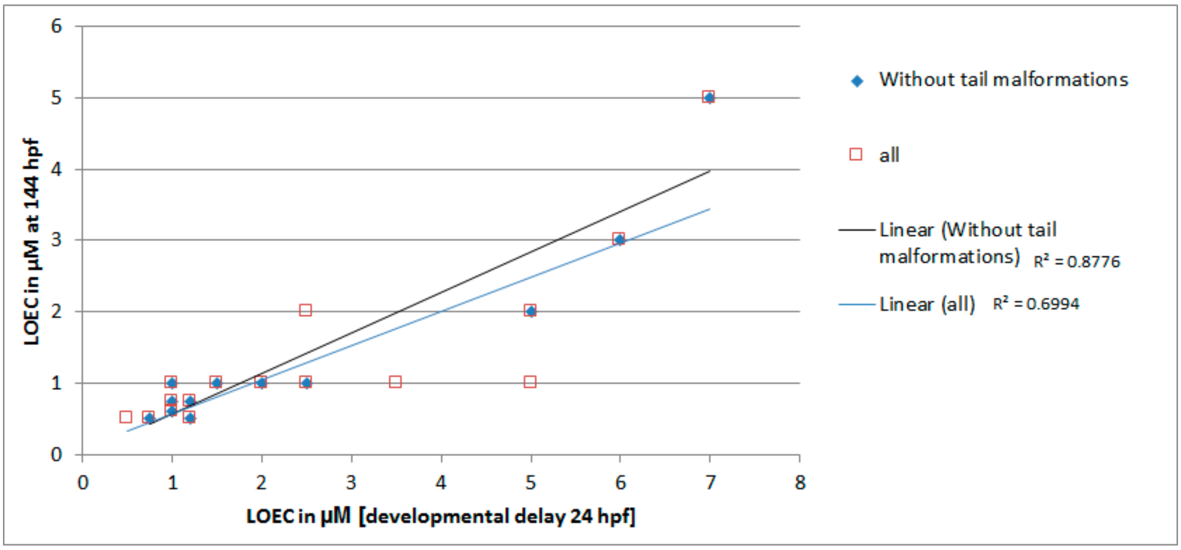

Figure S2. Correlation of LOECs derived from 144 hpf vs. LOECs derived from 24 hpf. With and without tail malformations (effects on heart beat, edema).

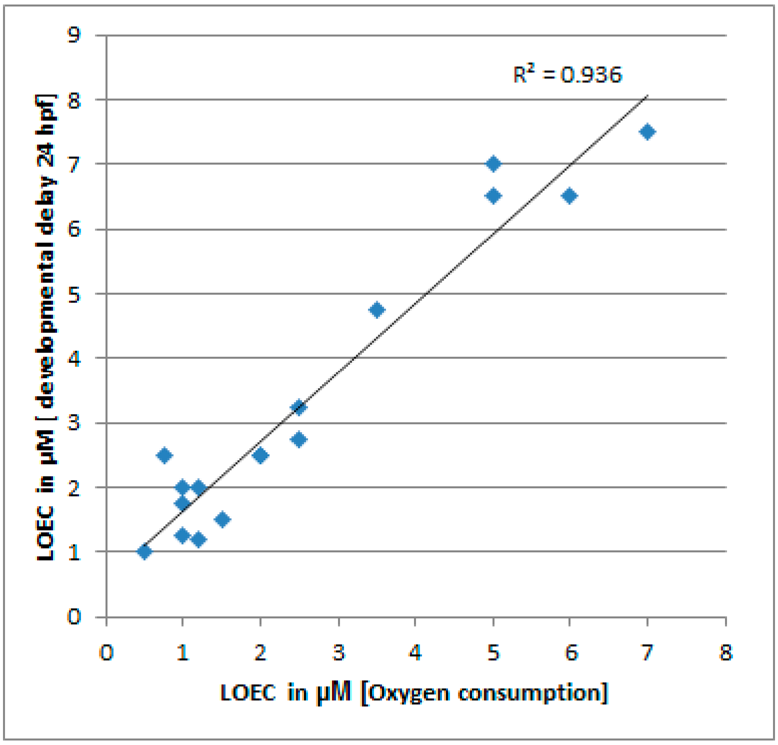

Figure S3. Correlation of LOECs derived from altered oxygen consumption versus LOECs derived from 24 hpf developmental delay.

Table S1. Effects observed at 6 days of development to LOEC and higher. Tail malformations are highlighted in bold.

Compound	Effects Observed at 144 hpf
2′-OH-6′-CL-BDE68	Delay/Stop in development (less pigmentation)—lower heartbeat
2-OH-BDE123	Delay/Stop in development
2′-OH-BDE28	Delay/Stop in development (less pigmentaion)
2′-OH-BDE66	Delay in development (no pigmentation) —cardiac edema
2′-OH-BDE68	Delay/Stop in development (less pigmentation)
3-OH-BDE153	Delay/Stop in development (less pigmentaion), **curved tail**
3′-OH-BDE154	Delay/Stop in development (less pigmentaion)
3-OH-BDE155	Delay/Stop in development (less pigmentaion), **curved tail**
3-OH-BDE47	Delay in development (less pigmentation), less heartbeat and movement
5-OH-BDE47	Delay/Stop in development, **curved tail**, delay in hatching, less movement
6-OH-5-CL-BDE47	Delay/Stop in development (less pigmentaion)
6-OH-BDE137	Delay/Stop in development (less pigmentation) less movement and a decreased heartbeat rate
6-OH-BDE47	Delay in development (less pigmentaion), **curved tail**, less heartbeat
6′-OH-BDE49	shorter, cardiac edema, less movement
6-OH-BDE85	Delay/Stop in development (less pigmentation)
6-OH-BDE90	**Tail malformations**, less movement
6-OH-BDE99	Delay in development (no pigmentation)—cardiac edema

Table S2. Blue mussel mix. Concentration of seven OH-PBDEs reported in Blue mussel in the concentrated and the diluted mixture (100× to 100×). Concentrations are in µM. NOEC and LOEC values for developmental toxicity in the first 24 h exposure are also shown.

	NOEC	LOEC	100× Mix	10× Mix	Blue Mussel Conc.	10× Mix	100× Mix
6-OH-BDE47	0.1	0.5	1.71	0.171	0.0171	0.00171	0.000171
2′-OH-BDE68	0.5	1	0.8	0.08	0.008	0.0008	0.00008
6-OH-BDE85	0.5	1	1.97	0.197	0.0197	0.00197	0.000197
6-OH-BDE90	0.25	2	0.84	0.084	0.0084	0.00084	0.000084
6-OH-BDE99	0.5	1	2.24	0.224	0.0224	0.00224	0.000224
2′-OH-BDE123	1.25	2	0.12	0.012	0.0012	0.00012	0.000012
6-OH-BDE137	0.1	1.2	1.08	0.108	0.0108	0.00108	0.000108
				below NOEC			
				above NOEC, below LOEC			
				above LOEC			

Table S3. Nominal and measured concentrations of 6-OH-BDE47 measured in exposure medium before and after 0–24 and 24–48 h in nM.

Nominal Conc. (nM)	Exposure Time (h)	Replicate 1 (nM)	Replicate 2 (nM)	Replicate 3 (nM)	Mean (nM)
75	0	8.8	8.9	8.2	8.6
75	24	1.1	1.8	1.7	1.5
75	48	n.a	0.9	1.0	1.0
100	0	12	13	11	12
100	24	2.9	2.9	2.8	2.8
100	48	2.3	1.9	2.2	2.1
140	0	15	15	14	15
140	24	2.9	3.3	3.6	3.3
140	48	3.0	n.a	2.1	2.5

n.a = not analysed.
